# Optimal Treatment Strategies for Pulmonary Large Cell Neuroendocrine Carcinoma Based on Molecular Subtypes

**DOI:** 10.3390/jcm15020619

**Published:** 2026-01-12

**Authors:** Hakan Yücel, Tülay Kuş, Sibel Cangi, Gökmen Aktaş

**Affiliations:** 1 Department of Medical Oncology, School of Medicine, Gaziantep University, Gaziantep 27410, Turkey; drtulaykus83@hotmail.com; 2 Department of Pathology, School of Medicine, Gaziantep University, Gaziantep 27410, Turkey; sibelcangi@hotmail.com; 3 Gaziantep Medical Point Private Hospital, Gaziantep 27310, Turkey; aktas_gokmen@hotmail.com

**Keywords:** pulmonary large cell neuroendocrine carcinoma, sclc, nsclc, chemotherapy, molecular subtype

## Abstract

**Background:** Pulmonary large-cell neuroendocrine carcinoma (LCNEC) is an uncommon and aggressive tumor for which the most effective systemic therapy remains uncertain. In metastatic LCNEC, chemotherapy approaches typically alternate between small-cell lung cancer (SCLC)-like and non-small-cell lung cancer (NSCLC)-like regimens. Emerging data indicate that treatment selection may be optimized through molecular subtype classification. This study aimed to evaluate the outcomes of SCLC-like and NSCLC-like chemotherapy (CT) regimens in relation to LCNEC molecular subtypes. **Methods:** This retrospective analysis included all patients diagnosed with LCNEC at Gaziantep University between January 2010 and October 2024. Individuals with available tumor tissue and complete clinical data were enrolled. LCNEC cases were categorized as SCLC-subtype or NSCLC-subtype according to the presence of *TP53* and *RB1* alterations. Platinum combined with etoposide, irinotecan, or topotecan was defined as SCLC-like CT, whereas platinum with taxanes or gemcitabine was considered NSCLC-like CT. Survival outcomes of both treatment types were compared across molecular subgroups using the Kaplan–Meier method. **Results:** Sixty-one patients met the inclusion criteria. The median overall survival (mOS) was 11.0 months (95% CI: 6.3–15.7). No significant difference in mOS was observed between SCLC-like and NSCLC-like regimens in the total cohort. When stratified by molecular subtype, patients with the SCLC subtype who received SCLC-like CT showed a longer mOS compared to those treated with NSCLC-like CT (15 [9.9–20.1] vs. 6 [3.9–8.1] months, respectively; *p* = 0.47), although this difference did not reach statistical significance. **Conclusions:** These findings suggest that molecular subclassification may help inform the choice of optimal systemic therapy in patients with LCNEC.

## 1. Introduction

Lung neuroendocrine (NE) tumors constitute approximately 20% of all lung cancers [1]. Based on their grade and level of differentiation, pulmonary neuroendocrine neoplasms (NENs) are classified into four categories: typical carcinoid (TC), atypical carcinoid (AC), small-cell lung carcinoma (SCLC), and large cell neuroendocrine carcinoma (LCNEC). Broadly, lung NENs are divided into two main groups according to their biological behavior and treatment approaches: neuroendocrine tumors (NETs) and neuroendocrine carcinomas (NECs). TC and AC, representing low-grade, well-differentiated lesions, fall under NETs, whereas NECs comprise high-grade, poorly differentiated carcinomas, including SCLC and LCNEC [2].

These two high-grade entities are primarily distinguished by their cytomorphologic features. LCNEC typically consists of large cells with abundant cytoplasm, coarse nuclear chromatin, and prominent nucleoli, while SCLC is composed of smaller cells with scant cytoplasm, finely granular chromatin, and inconspicuous nucleoli [2]. Pulmonary LCNEC is exceedingly rare, accounting for less than 3% of all lung cancers worldwide [3]. First described as a distinct pathological entity by Travis et al., LCNEC has been categorized by the World Health Organization (WHO) since 2015 within the spectrum of high-grade, poorly differentiated neuroendocrine carcinomas alongside SCLC [4,5].

Clinically, LCNEC shares several characteristics with SCLC, including aggressive behavior, strong correlation with tobacco exposure, predominance in males, older age at presentation, frequent metastatic disease, and overall poor prognosis. Pathologically, both exhibit high mitotic activity, necrosis, and positive neuroendocrine immunohistochemical staining [6]. Because of its rarity and overlap with SCLC in both morphology and clinical course, therapeutic strategies for LCNEC have often been extrapolated from SCLC regimens, primarily derived from small retrospective studies [7]. Nonetheless, LCNEC is characterized by substantial genomic heterogeneity, encompassing tumors with either classical SCLC-type alterations or NSCLC-like genomic profiles combined with neuroendocrine differentiation, making standardized treatment selection particularly challenging [8].

Rekhtman et al. first identified two distinct genomic subtypes of LCNEC through next-generation sequencing (NGS): an SCLC-like subtype (≈40%) harboring concurrent alterations in *TP53* and *RB1*, and an NSCLC-like subtype (≈56%) lacking co-alteration of these genes [9]. The NSCLC-like subtype may also feature mutations typical of adenocarcinoma, such as *STK11*, *KRAS*, and *KEAP1* [7]. A much rarer “carcinoid-like” LCNEC subgroup (≈4%) lacks both *RB1* and *TP53* alterations. According to the 2021 WHO Classification of Thoracic Tumours, *RB1* and *TP53* immunohistochemical (IHC) expression can be used as a surrogate for molecular subclassification when NGS is not available; however, its application in routine clinical decision-making has not yet been recommended [2].

Although limited data from small cohorts suggest that molecular subtyping may help tailor systemic therapy according to SCLC-like or NSCLC-like patterns [10], robust clinical evidence is still lacking. Consequently, treatment strategies remain heterogeneous and non-standardized. The present study aims to assess the therapeutic efficacy of SCLC-based and NSCLC-based chemotherapy regimens in LCNEC patients stratified by molecular subtype.

## 2. Materials and Methods

### 2.1. Study Design, Patient Selection and Study Objective

This single-center, observational, retrospective study was conducted by the Department of Medical Oncology at Gaziantep University. Patients diagnosed with LCNEC between January 2010 and October 2024 were retrospectively screened. Eligible patients were aged over 18 years, had stage I–IV LCNEC, and had available tumor tissue suitable for mutational analysis. Tumor staging was determined according to the American Joint Committee on Cancer (AJCC) 8th Edition (2017).

The following variables were collected: age, sex, Eastern Cooperative Oncology Group (ECOG) performance status (PS), smoking history, disease stage (1: stage I–IIA; 2: stage IIB–III; 3: stage IV), history of surgery, chemotherapy (CT), or radiotherapy (RT), metastatic site(s), number of metastatic sites, details of first- and subsequent-line CT regimens, and dates of diagnosis, treatment initiation, disease progression, and death.

The primary objective of the study was to evaluate the therapeutic impact of SCLC-like and NSCLC-like chemotherapy regimens on LCNEC by stratifying patients into SCLC-like and NSCLC-like pathological subtypes.

The study was performed in accordance with the ethical principles outlined in the Declaration of Helsinki and approved by the Ethics Committee of Gaziantep University Faculty of Medicine (Approval No: 416).

### 2.2. Pathological Evaluation for Molecular Subtyping: SCLC-Subtype vs. NSCLC-Subtype

Haematoxylin and eosin (H&E)-stained slides from resected tumors were reviewed by a thoracic pathologist (S.C.) and selected for immunohistochemical analysis. Serial 4 μm-thick sections from formalin-fixed, paraffin-embedded (FFPE) tissue samples were mounted on adhesive slides and stained for Rb1 (mouse, clone 13A10; Novocastra, Leica Biosystems, Wetzlar, Germany), Ki-67 (monoclonal mouse, clone 30-9; Ventana, Roche Diagnostics, Tucson, AZ, USA), and p53 (mouse, clone DO-7; DAKO, Agilent Technologies, Santa Clara, CA, USA). Immunostaining was performed using the Ventana Benchmark ULTRA platform (Roche Diagnostics, Basel, Switzerland). Samples were considered assessable when sufficient tumor tissue was available for interpretation.

RB1 expression was classified as mutant when nuclear staining was absent in ≥90% of tumor cells and as wild-type when variable nuclear positivity was present.

p53 expression was categorized into three groups: overexpression (≥80% strong diffuse nuclear positivity), complete absence (negative in ≥90% of cells), and wild-type (heterogeneous nuclear expression).

The Ki-67 proliferation index was calculated from the most active (“hot spot”) area on each slide.

A diagnosis of LCNEC was confirmed based on the 2021 World Health Organization (WHO) Classification of Lung Tumors [2], which requires neuroendocrine morphology, a high mitotic index, and positivity for at least one neuroendocrine marker (CD56, chromogranin A, or synaptophysin).

LCNEC tumors were subclassified as SCLC-like or NSCLC-like according to immunostaining patterns: tumors with concurrent alterations in *TP53* and *RB1* were defined as the SCLC-like subtype, while those lacking co-alterations were categorized as the NSCLC-like subtype [4].

### 2.3. Treatments: SCLC-Like

Chemotherapy regimens consisting of platinum combined with etoposide, irinotecan, or topotecan were defined as SCLC-like CTs.

Regimens combining platinum with vinorelbine, gemcitabine, or a taxane were classified as NSCLC-like CTs.

### 2.4. Statistical Analysis

Categorical variables were summarized as frequencies and percentages, while continuous variables were expressed as means ± standard deviations (SD). Comparisons between categorical variables were conducted using Fisher’s exact or Chi-square tests, as appropriate.

Progression-free survival (PFS) was defined as the interval between the initiation of chemotherapy and either documented disease progression or the last follow-up. Overall survival (OS) was defined as the time from chemotherapy initiation to death or last follow-up. Survival outcomes were estimated using the Kaplan–Meier method, and intergroup differences in PFS and OS were compared with the log-rank test.

Variables with a *p*-value < 0.1 in univariate analysis were entered into multivariate Cox proportional hazards models for both PFS and OS. A *p*-value ≤ 0.05 was considered statistically significant.

All analyses were performed using IBM SPSS Statistics, version 22.0 (IBM Corp., Armonk, NY, USA).

## 3. Results

### 3.1. The Demographic, Clinical, and Pathological Features of the Patients and the Distribution of Treatments

All patients diagnosed with LCNEC at our institution between January 2010 and October 2024 were consecutively evaluated. Those lacking pathological confirmation or adequate clinical/radiological follow-up were excluded. A total of 61 patients were eligible for survival analysis. However, genomic profiling could not be performed in 5 cases due to insufficient tumor tissue, and 5 patients declined chemotherapy. One patient received targeted therapy. Consequently, 50 patients were included in the final analysis, in which treatment regimens were compared based on molecular classification.

The mean patient age was 63.2 ± 8.9 years. Of the cohort, 90.2% (*n* = 55) were male, and 9.8% (*n* = 6) were female. Among the 39 patients with available smoking data, 92.3% (*n* = 36) had a smoking history exceeding 20 pack-years.

Early-stage cases were managed with localized modalities (surgery and/or radiotherapy). There were 46 metastatic patients (75.4%), while the remaining were diagnosed at stage I–III.

A total of 27 (44.3%), 20 (32.8%), and 9 (14.7%) patients received first-, second-, and third-line chemotherapy (CT), respectively.

For first-line therapy, SCLC-like regimens were more frequently administered than NSCLC-like regimens (88.9% vs. 11.1%). In subsequent treatment lines, the proportions of SCLC-like and NSCLC-like CT use were approximately comparable.

Detailed demographic, clinical, and pathological features of the entire cohort are summarized in Table 1.

Among patients included in the molecular analysis, 35 (62.5%) were classified as the SCLC-subtype and 21 (37.5%) as the NSCLC-subtype. The immunohistochemical (IHC) staining profiles of both groups were largely similar; however, as expected, the proportion of tumors with Ki-67 ≥ 80% was lower in the NSCLC subtype.

Tumor stage distribution did not differ significantly between SCLC- and NSCLC-subtypes (Stage I–IIA: 11.4% vs. 4.8%; Stage IIB–III: 20% vs. 9.5%; Stage IV: 68.6% vs. 85.7%; *p* = 0.208).

As shown in Table 1, other clinical and pathological characteristics were also comparable between the two molecular subgroups.

At the time of the last follow-up, 78.7% of patients (48/61) had died. In the overall cohort, the median progression-free survival (PFS) was 5.0 months (95% CI: 3.8–6.3), and the median overall survival (mOS) was 11.0 months (95% CI: 6.3–15.7).

Among the treated patients, 48 received SCLC-like chemotherapy regimens, while 6 were administered NSCLC-like regimens as first-line therapy.

**Figure 1:**
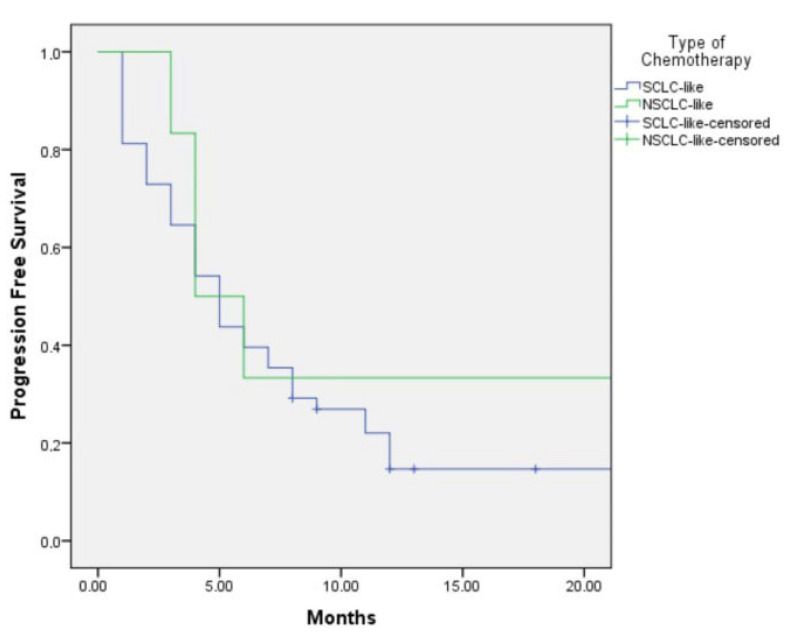
The Kaplan–Meier Graph of progression-free survival according to SCLC-like or NSCLC-like chemotherapy.

The median PFS was 5.0 months (95% CI: 3.6–6.4) in the SCLC-like treatment group and 4.0 months (95% CI: 1.6–6.4) in the NSCLC-like group. This difference was not statistically significant (*p* = 0.48) (Figure 1).

**Figure 2:**
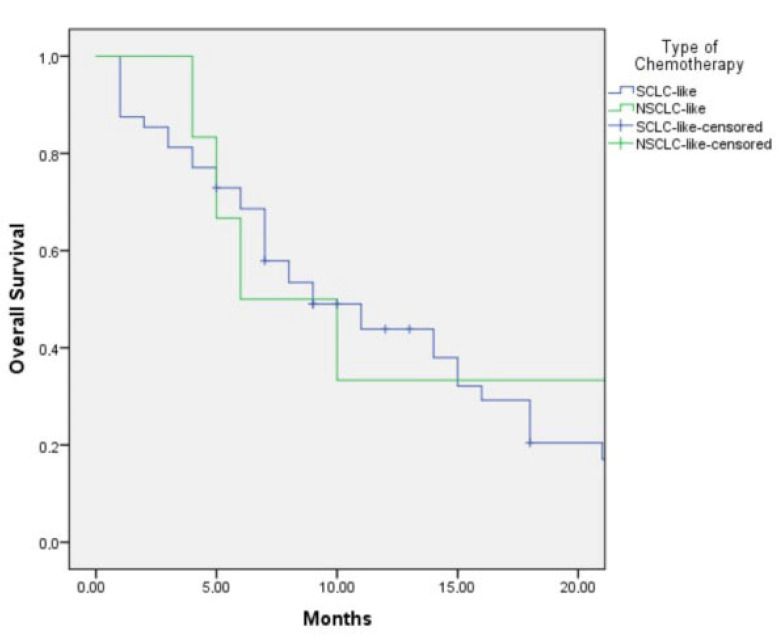
The Kaplan–Meier Graph of overall survival according to SCLC-like or NSCLC-like chemotherapy.

The median overall survival (OS) was 9.0 months (95% CI: 4.9–13.1) in patients who received SCLC-like regimens and 6.0 months (95% CI: 2.0–23.0) in those treated with NSCLC-like regimens (*p* = 0.402) (Figure 2). Although SCLC-like chemotherapy demonstrated a numerically longer OS, the difference did not reach statistical significance.

Continuation of chemotherapy beyond the first line was associated with longer median survival times—4.0 months (95% CI: 1.5–6.5) for first-line, 11.0 months (95% CI: 4.4–17.5) for second-line, and 14.0 months (95% CI: 2.3–25.7) for third-line therapy—although this trend did not reach statistical significance (*p* = 0.92). Following disease progression, 13 patients received SCLC-like chemotherapy (CT) and another 13 received NSCLC-like CT as second-line treatment. While SCLC-like CT in the second-line setting yielded a numerically longer overall survival (14.0 [8.0–19.9] vs. 11.0 [3.9–18.0] months), the difference was not statistically significant (*p* = 0.247). None of the patients received immunotherapy, as LCNEC is not currently included among the indications recommended by the NCCN guidelines. One patient harboring an *ALK* fusion received targeted therapy with an ALK inhibitor.

Among clinicopathological variables, disease stage and patient age were significant factors influencing both progression-free survival (PFS) and overall survival (OS), as presented in Table 2. No significant associations were observed between OS and other clinical or pathological parameters, including molecular subtype (SCLC-like vs. NSCLC-like) and the number or distribution of metastatic sites.

As summarized in Table 2, the presence of liver metastases was correlated with poorer PFS, whereas other variables did not show a statistically significant relationship with PFS.

### 3.2. Effect of Chemotherapy Regimens on Survival by Pathological SCLC-Subtype and NSCLC-Subtype

The analysis included patients who underwent post-treatment response assessment.

**Figure 3:**
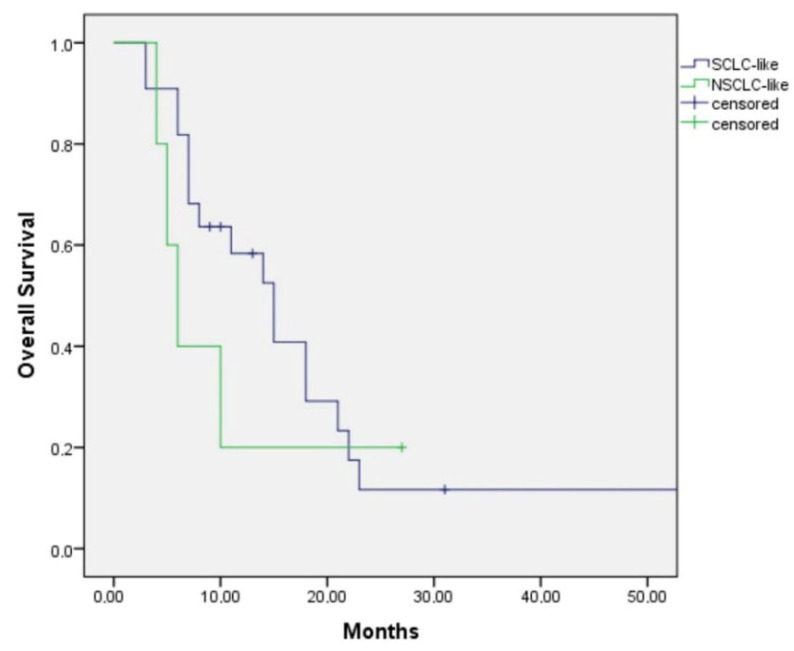
Kaplan–Meier Graph of the patients with SCLC-subtype LCNEC according to SCLC-like chemotherapy or NSCLC-like chemotherapy at first line.

Among those with the SCLC-like subtype, patients treated with first-line SCLC-like chemotherapy had a median overall survival (OS) of 15.0 months (95% CI: 9.9–20.1), whereas those receiving NSCLC-like chemotherapy had a median OS of 6.0 months (95% CI: 3.9–8.1) (*p* = 0.47) (Figure 3). Although the survival duration was numerically longer in patients treated with SCLC-like regimens, the difference did not reach statistical significance.

For the NSCLC-like subtype, all patients received SCLC-like chemotherapy, yielding a median OS of 9.0 months. As no patients in this group received NSCLC-like chemotherapy, the comparative efficacy of NSCLC-like regimens in the first-line setting could not be evaluated.

**Figure 4:**
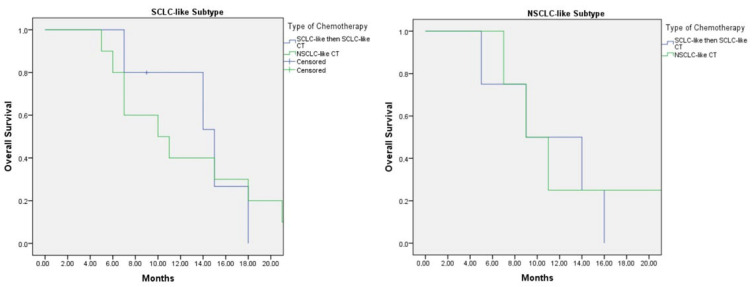
Kaplan–Meier Graph of overall survival of SCLC-like subtype according to first and second line type of CT (**Left**). Kaplan–Meier Graph of overall survival of NSCLC-like subtype according to first and second line type of CT (**Right**).

Among patients who received second-line chemotherapy following disease progression, five with the SCLC-like subtype were treated with SCLC-like chemotherapy in both the first- and second-line settings, while 15 patients received NSCLC-like chemotherapy either as first-line or subsequent-line therapy. In this group, the median overall survival (OS) was 15.0 months (95% CI: 8.4–21.6) for patients treated with SCLC-like chemotherapy in both lines, compared with 10.0 months (95% CI: 3.8–16.2) for those who received NSCLC-like chemotherapy at any line (*p* = 0.89) (Figure 4).

For patients with the NSCLC-like subtype, the median OS was 9.0 months (95% CI: 1.0–17.8) in those treated with two lines of SCLC-like chemotherapy, which was comparable to a median OS of 9.0 months (95% CI: 5.0–12.9) in those who received NSCLC-like chemotherapy at any line (*p* = 0.750) (Figure 4).

## 4. Discussion

The genetic alterations underlying LCNEC have not yet been fully characterized. Reported mutation frequencies indicate that *TP53* alterations occur in 46.4–92% of cases, whereas *RB1* mutations are observed in approximately 26–42%. Co-mutations of both genes, which define the pathological SCLC-like subtype, are present in about 40% of LCNEC cases [11,12]. In the present study, *RB1* and *TP53* co-mutations were detected in 57.4% of patients, aligning with previously reported ranges.

This retrospective analysis of 61 patients with advanced LCNEC aimed to identify optimal therapeutic strategies according to molecular subtypes. Patients were classified as either SCLC-like or NSCLC-like based on immunohistochemical profiles, and outcomes were evaluated according to the type of chemotherapy regimen administered (SCLC-like vs. NSCLC-like). Among patients with the SCLC-like subtype, those treated with first-line SCLC-like chemotherapy achieved a median overall survival (OS) of 15.0 months (95% CI: 9.9–20.1), compared with 6.0 months (95% CI: 3.9–8.1) in those who received NSCLC-like regimens. Although this difference favored SCLC-like chemotherapy, statistical significance was not reached (*p* = 0.47), likely due to the limited sample size (Figure 1).

In contrast, all patients with the NSCLC-like subtype received SCLC-like chemotherapy, resulting in a median OS of 9.0 months. Consequently, the efficacy of NSCLC-like chemotherapy could not be assessed in this group. The modest OS observed with SCLC-like regimens suggests a need to explore alternative treatment strategies for NSCLC-like LCNEC. Comprehensive molecular profiling and identification of actionable alterations may offer opportunities for targeted therapy and improved survival in these patients.

In the subgroup of patients who received second-line chemotherapy following progression, those with the SCLC-like subtype treated with SCLC-like chemotherapy in both the first and second lines achieved a median OS of 15.0 months (95% CI: 8.4–21.6). This was numerically longer than the OS of 10.0 months (95% CI: 3.8–16.2) observed in patients who received NSCLC-like chemotherapy at any treatment line (*p* = 0.89). Although the difference was not statistically significant, the trend suggests a potential benefit from maintaining SCLC-like regimens in SCLC-like molecular subtypes.

For the NSCLC-like subtype, the median OS was 9.0 months (95% CI: 1.0–17.8) in those who received two lines of SCLC-like chemotherapy, comparable to 9.0 months (95% CI: 5.0–12.9) in patients treated with NSCLC-like chemotherapy at any line (*p* = 0.750). While patients with the NSCLC-like subtype demonstrated lower survival rates than those with the SCLC-like subtype, SCLC-like chemotherapy did not appear to adversely affect outcomes when compared with NSCLC-like regimens.

LCNEC is characterized by poor survival outcomes, even in early-stage disease, with a reported 5-year survival rate of only 33% among stage I patients [13]. Several retrospective analyses have demonstrated that the addition of chemotherapy to surgical management significantly improves survival in LCNEC [12,13]. For example, platinum plus etoposide, representing an SCLC-like chemotherapy (CT) regimen, has been associated with superior overall survival (OS) compared with surgery alone (5-year OS: 64.5% vs. 48.4%, *p* < 0.001) [14].

In our study, five patients (8.9%) were diagnosed at an early stage and underwent curative-intent surgery. The median OS for these patients was not reached during follow-up. Of these, four were classified as SCLC-subtype and one as NSCLC-subtype, and all received adjuvant SCLC-like chemotherapy.

The optimal chemotherapy approach for advanced LCNEC remains uncertain. Standard regimens used for NSCLC and SCLC are commonly adopted, yet their effectiveness appears reduced when applied to LCNEC [15]. The prospective, multicenter, single-arm phase II GFPC 0302 trial investigated the combination of platinum and etoposide in 42 patients with LCNEC, reporting a median progression-free survival (PFS) of 5.2 months (95% CI: 3.1–6.6) and a median OS of 7.7 months (95% CI: 6.0–9.6) [16].

While some small retrospective series have suggested that platinum–etoposide regimens yield better outcomes than NSCLC-like chemotherapy, other studies have reported contradictory findings [7].

The Netherlands Cancer Registry, in conjunction with the Netherlands Pathology Registry (PALGA), evaluated outcomes in LCNEC patients treated with either NSCLC-like or SCLC-like chemotherapy. The study demonstrated that patients receiving NSCLC-like regimens achieved a median OS of 8.5 months (95% CI: 7.0–9.9), significantly longer than the 6.7 months (95% CI: 5.0–8.5) observed in those treated with SCLC-like regimens (HR: 1.66, 95% CI: 1.08–2.56; *p* = 0.020) [17].

Following molecular studies that defined two major genomic subgroups of LCNEC—the SCLC-like and NSCLC-like subtypes—treatment decisions guided by molecular characteristics have gained increasing attention.

Derks et al. conducted a retrospective comparison of platinum–etoposide (SCLC-PE) versus platinum/taxane–gemcitabine (NSCLC-GEM/TAX) regimens in LCNEC patients, taking molecular subtypes into account. In their analysis, patients were stratified as *RB1*-mutated or *RB1* wild-type to evaluate the predictive value of molecular classification for chemotherapy outcomes. Individuals with wild-type *RB1* or preserved *RB1* protein expression demonstrated improved survival with NSCLC-GEM/TAX regimens compared to those treated with SCLC-PE chemotherapy. In contrast, no survival difference was observed among *RB1*-mutated patients or those lacking *RB1* protein expression [18].

In that study, molecular subclassification was based solely on *RB1* mutation status, with most cases also harboring *TP53* mutations. However, current recommendations emphasize the use of concurrent *RB1* and *TP53* co-mutations to define the SCLC-like molecular subtype of LCNEC.

In our cohort, molecular subtyping was performed according to this dual-mutation model: tumors with co-mutations in *RB1* and *TP53* were categorized as SCLC-like, whereas those without concurrent alterations were considered NSCLC-like.

Among patients with *RB1*-mutated SCLC-like LCNEC, continuation of SCLC-like chemotherapy across both first- and second-line settings was associated with longer survival, though the difference did not reach statistical significance. For tumors with wild-type *RB1*, a comparison between SCLC-like and NSCLC-like regimens in the first-line setting could not be performed due to limited data, and no significant differences were observed in the second-line setting.

The discrepancies between our findings and those of Derks et al. may stem from differences in molecular classification criteria and the relatively small sample sizes of both studies.

In another case series, Zhuo et al. evaluated the prognostic and therapeutic relevance of genomic subtyping in 45 patients with LCNEC [19]. Consistent with our classification approach, patients harboring concurrent *RB1* and *TP53* mutations were defined as SCLC-like LCNEC, while those lacking co-alterations in these genes were categorized as NSCLC-like LCNEC. Similarly to our findings, the combination of platinum and etoposide was associated with superior overall survival (OS) in the SCLC-like subtype compared with pemetrexed–platinum or gemcitabine/taxane–platinum doublets. Moreover, in the NSCLC-like subtype, NSCLC-based chemotherapy regimens were linked to shorter OS compared with platinum–etoposide therapy [19].

In a separate study involving eight tumor samples with *TP53* mutations, *RB1* loss of function—accompanied by *MYC/MYCN* amplification and *NOTCH* family (*NOTCH1/2/4*) mutations—was identified as a predictor of the SCLC-like subtype [10]. These observations suggest that molecular alterations beyond *TP53–RB1* co-mutations may contribute to the more precise identification of the SCLC-like subgroup. Although that study included detailed molecular profiling in only 12 patients, the findings underscore the necessity of integrating additional genomic markers to improve subtype characterization.

Our results align with these data, further supporting the role of SCLC-like chemotherapy in patients with the SCLC-like molecular subtype. While the magnitude of benefit appeared greater in earlier studies, the consistent trend reinforces the therapeutic relevance of molecular subtyping in LCNEC.

The main limitation of this study is its relatively small sample size and retrospective nature, which is consistent with other reports in the literature. However, given the rarity of LCNEC, conducting large-scale prospective trials remains challenging. Existing publications are limited to a few small-cohort studies, underscoring the importance of aggregating multicenter data to improve understanding of this uncommon malignancy.

In the present study, 61 patients with LCNEC were evaluated, and sufficient tumor tissue for molecular subgrouping was available in 50 cases—a comparatively robust sample size relative to similar investigations. Our results suggest that assessment of *TP53* and *RB1* mutation status plays a key role in defining LCNEC molecular subtypes and may help guide chemotherapy selection.

For patients classified as NSCLC-like, more comprehensive molecular profiling is warranted to identify actionable targets and refine treatment strategies, as this subgroup exhibits poorer outcomes with conventional chemotherapy. Conversely, in patients with the SCLC-like subtype, continuation of SCLC-like chemotherapy across both first- and second-line settings was associated with improved survival compared to switching to NSCLC-like regimens.

## 5. Conclusions

Molecular subtyping represents a key determinant in selecting the most appropriate chemotherapy regimen for LCNEC, underscoring the clinical importance of integrating genomic classification into therapeutic decision-making.

## Figures and Tables

**Table 1:** Comparison of the clinicopathological features of the patients with SCLC-subtype and NSCLC-subtype.

	N (%) All Patients	SCLC-Subtype (N = 35; 57.4%)	NSCLC-Subtype (N = 21; 34.4%)	*p* Value
Age				
<65	32 (52.5)	20 (57.1)	9 (42.9)	0.30
≥65	29 (47.5)	15 (42.9)	12 (57.1)	
Age, mean	63.2 (±8.9)	63.4 (±9.0)	63.7 (±8.5)	0.87
Gender				
Female	6 (9.8)	2 (5.7)	2 (9.5)	0.63
Male	55 (90.2)	33 (94.3)	19 (90.5)	
Smoking history, packs/year	50	52.0	59.9	0.39
Stage				
I-IIA	5 (8.2)	4 (11.4)	1 (4.8)	0.36
IIB-III	10 (16.4)	7 (20.0)	2 (9.5)	
IV	46 (75.4)	24 (68.6)	18 (85.7)	
Pathological features				
TTF staining (+)	24 (42.1)	17 (50.0)	6 (30.0)	0.17
Chromogranin staining (+)	35 (57.4)	21 (67.7)	12 (66.7)	1.00
Synaptophysin staining (+)	40 (78.4)	24 (75.0)	14 (87.5)	0.27
Ki-67 ≥ 80	46 (75.4)	28 (80.0)	14 (66.7)	0.21
Ki-67, mean	79.3 (±8.4)	79.7 (±7.8)	77.4 (±9.2)	0.16
Number of metastatic sites				
0	15 (24.6)	11 (31.4)	3 (14.3)	0.25
1	27 (44.3)	13 (37.1)	12 (57.1)	
≥2	19 (31.1)	11 (31.4)	6 (28.6)	
Presence of CNS metastasis	10 (16.4)	5 (14.3)	3 (14.3)	1.00
Presence of liver metastasis	9 (14.8)	5 (17.2)	3 (17.6)	1.00
Presence of bone metastasis	11 (18.0)	6 (20.7)	4 (23.5)	1.00
Treatment line				
1	27 (44.3)	16 (50.0)	10 (50.0)	0.64
2	20 (32.8)	10 (31.2)	8 (40.9)	
≥3	9 (14.7)	6 (18.8)	2 (10.0)	
Treatment type (*n* = 54)				
SCLC-like CTs	48 (88.9)	27 (84.4)	18 (100)	0.145
NSCLC-like CTs	6 (11.1)	5 (15.6)	0	
Second-line therapy type (*n* = 26)				
SCLC-like CTs	13 (50)	7 (46.7)	4 (50.0)	1.00
NSCLC-like CTs	13 (50)	8 (53.3)	4 (50.0)	

SCLC: Small-cell lung cancer; NSCLC: Non-small-cell lung cancer; CTs: Chemotherapies.

**Table 2:** Progression-Free Survival and Overall Survival Data Based on Clinicopathological Parameters and Chemotherapy Regimens.

	PFS Hazard Ratio; 95%CI	*p* Value	OS Hazard Ratio; 95%CI	*p* Value
Age				
<65	7.0 (1.6–12.4)	0.003	16.0 (10.1–21.9)	<0.001
≥65	4.0 (3.1–4.9)		5.0 (3.5–6.5)	
Gender				
Female	3.0 (0.0–6.6)		3.0 (1.0–7.8)	0.05
Male	5.0 (3.3–6.7)	0.50	10.0 (6.7–13.3)	
Stage				
I-IIA	Non-reached	<0.001	Non-reached	<0.001
IIB-III	12.0 (0.0–28.9)		21.0 (6.9–35.1)	
IV	4.0 (2.9–6.1)		7.0 (4.9–9.1)	
Pathological features				
TTF staining (−) vs. (+)	5.0 (3.1–6.9) vs. 5.0 (2.6–7.4)	0.87	9.0 (5.3–12.7) vs. 11.0 (2.7–19.3)	0.76
Chromogranin staining (−) vs. (+)	8.0 (2.1–13.8) vs. 4.0 (2.6–5.4)	0.018	14.0 (2.0–26.0) vs. 10.0 (6.6–13.4)	0.35
Synaptophysin staining (−) vs. (+)	6.0 (3.9–8.1) vs. 5.0 (3.4–6.5)	0.89	7.0 (1.6–12.9) vs. 10.0 (6.7–13.2)	0.79
Ki-67 ≥ 80 vs. <80	6.0 (2.2–9.7) vs. 4.0 (2.5–5.4)	0.48	11.0 (4.1–17.9) vs. 8.0 (5.4–10.7)	0.69
Pathological subtype				
SCLC-subtype	5.0 (3.7–6.6)		11.0 (5.5–16.5)	0.362
NSCLC-subtype	4.0 (1.7–6.2)	0.57	9.0 (5.2–12.8)	
Treatment type				
SCLC-like CTs	5.0 (3.6–6.4)		9.0 (4.9–13.11)	0.402
NSCLC-like CTs	4.0 (1.6–6.4)	0.48	6.0 (1.0–12.0)	
Second-line therapy type				
SCLC-like CTs	-		14.0 (8.0–19.9)	0.247
NSCLC-like CTs			11.0 (3.9–18.0)	
Number of metastatic sites				
1 vs. ≥2	4.0 (2.3–5.6) vs. 4.0 (2.9–5.0)	0.78	8.0 (5.6–10.4) vs. 7.0 (5.1–8.9)	0.88
Presence of CNS metastasis				
Absent vs. present	5.0 (3.2–6.7) vs. 4.0 (2.5–5.5)	0.17	10.0 (6.3–13.7) vs. 7.0 (4.1–9.8)	0.77
Presence of liver metastasis				
Absent vs. present	6.0 (3.5–8.5) vs. 4.0 (1.1–6.9)	0.040	10.0 (6.5–13.4) vs. 7.0 (4.4–9.5)	0.18
Presence of bone metastasis				
Absent vs. present	5.0 (2.58–7.4) vs. 5.0 (2.7–7.3)	0.12	10.0 (6.7–13.2) vs. 5.0 (1.0–15.9)	0.15
Treatment line				
1	-		4.0 (1.5–6.5)	0.92
2			11.0 (4.4–17.5)	
≥3			14.0 (2.3–25.7)	

SCLC: Small-cell lung cancer; NSCLC: Non-small-cell lung cancer; CTs: Chemotherapies; PFS: Progression-free survival; OS: Overall survival.

## Data Availability

The original contributions presented in this study are included in the article. Further inquiries can be directed to the corresponding author.
